# Perceptions and Barriers to Screening Mammography and Clinical Breast Examination: A Survey Study of Underserved Populations in North Texas

**DOI:** 10.1002/puh2.70032

**Published:** 2025-02-06

**Authors:** Sofia Eva Olsson, Sameep Shah, Erin Haase, Emma Butler, Isabella Amado, Kelly Pagidas

**Affiliations:** ^1^ Anne Burnett Marion School of Medicine Texas Christian University Fort Worth Texas USA; ^2^ Department of Computer Science College of Science and Engineering Texas Christian University Fort Worth Texas USA; ^3^ School of Medicine Louisiana State University Health Shreveport Shreveport Louisiana USA

**Keywords:** barriers to care, breast cancer perceptions, breast cancer screening, clinical breast exam, mammography, preventative health, women's health

## Abstract

**Background:**

Mammography serves as the primary screening tool for detecting breast cancer, and clinical breast examination serves as an additional low‐risk technique. There are known socioeconomic disparities in screening accessibility that correlate with breast cancer mortality and tumor stage at diagnosis. Identifying patient barriers and sentiments is a vital step in increasing compliance rates amongst vulnerable populations.

**Methods:**

A link to a survey available in English and Spanish was distributed across zip codes with the lowest median household incomes in Fort Worth, Texas. Data collection took place between November 2022 and November 2023. Only female participants aged 18 or older were included in the study. Statistical analysis was performed on IBM SPSS, Python, and Pandas library softwares.

**Results:**

Hispanic, low‐income, and less educated individuals were more likely to have inadequate screening mammography and clinical breast examination status. This is due to several self‐reported barriers including cost, lack of knowledge, and lack of time. The majority of patients reported interest in receiving women's healthcare (67.3%), believed screening allowed for early breast cancer detection (72.7%), and believed screenings decreased breast cancer mortality (69.1%).

**Conclusions:**

There are clear discrepancies in access to breast cancer screenings despite a majority of respondents acknowledging their benefit. We suggest the aforementioned demographics be targeted in interventions to improve free or low‐cost access and education surrounding breast cancer screenings. This study would benefit from further data collection and expansion to multiple cities in the United States.

## Graphical abstract

The most common barrier to screening mammography and clinical breast examination in North Texas was cost. Individuals of non‐White race, income of 40,000 USD or lower, and a maximum education of high school or lower were more likely to have been overdue for screening mammography.

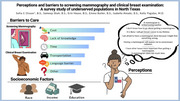

## 1 Introduction

Over the last decade, breast cancer incidence has risen at a rate of 0.5% each year; however, mortality has decreased at a rate of approximately 1.3% per year [1]. In fact, since 1989 in which breast cancer demonstrated peak mortality, death rate has dropped an estimated 43% [1]. The impressive decrease in mortality can likely be attributed to earlier detection following increased screening and awareness as states began requiring health insurance companies to cover screening mammography from 1987 to 2000 [2].

Mammography serves as the primary screening tool for detecting breast cancer. Although the United States Preventive Services Task Force (USPSTF) advocates for biennial screenings in women aged 50–74 [3], the American Cancer Society (ACS) recommends annual mammograms for women starting at age 40 [4]. The American College of Radiology (ACR) and Society of Breast Imaging (SBI) also recommend annual screening mammography beginning at age 40 with a risk assessment at age 25 [5]. Although mammography is the gold standard for breast cancer screening, clinical breast exams are a low‐risk, cost‐effective screening method, which can be started at an earlier age. This involves the manual palpation of the breasts by a trained clinician. The ACS does not recommend clinical breast examination due to unclear evidence regarding its efficacy; however, the American College of Obstetricians and Gynecologists (ACOG) endorse clinical breast exams in high‐risk patients and those aged 25 through 39 [4, 6]. ACOG highlights the necessity of shared decision making and patient preferences in this recommendation and notes that mammography with clinical breast examination detects 2%–6% more cases of invasive cancer than mammography alone [6]. Regardless, regular screenings play a pivotal role in early breast cancer detection, thereby mitigating morbidity and mortality risks [7].

There are known socioeconomic disparities in screening accessibility that correlate with breast cancer mortality and stage at diagnosis [1]. In Texas, only 68.2% of women aged 40 and above have undergone a mammogram within the last 2 years [8]. This suboptimal uptake can be attributed to various barriers such as financial constraints, limited access to healthcare services, insufficient awareness, and conflicting medical guidelines. Fort Worth, Texas, situated within one of the largest metropolitan areas in the United States (US), reflects a demographic composition closely aligned with the national averages [9, 10]. For example, the city has comparable figures in terms of disability rates, educational attainment, computer access, and poverty levels, aligning with broader national trends [9, 10]. However, a concerning disparity emerges in healthcare coverage, with 21% of Fort Worth residents lacking health insurance, higher than the national rate of 9.3% [9, 10]. Therefore, understanding barriers and perceptions of breast cancer screening in this region can provide valuable insight based on demographic similarities to the United States and an emphasis on vulnerable populations lacking healthcare.

Addressing healthcare disparities necessitates a targeted approach that identifies and addresses specific barriers hindering individuals from accessing screening services. By discerning the root causes of underutilization, interventions tailored to the unique needs of Fort Worth's population can be devised. Given the demographic similarities between North Texas and the broader United States, insights gleaned from local data hold considerable value in formulating strategies aimed at promoting preventive care utilization nationwide.

## 2 Methods

### 2.1 Data Collection

A survey inquiring about individual barriers and perceptions of screening mammography and clinical breast exams was designed on Qualtrics software. The survey was made available in both English and Spanish. A large group of research volunteers distributed English and Spanish flyers with a quick response code to the survey. They were placed with permission in public establishments located in the half of Fort Worth zip codes with lowest median household income as described by the 2017–2021 census [11]. Volunteers were educated to focus on flyer distribution at necessary, relatively low‐cost establishments (i.e., laundromat, local grocery store, community centers, and food pantries). Volunteers were educated to avoid luxury or nonessential establishments (restaurants, coffee shops, clothing outlets, and high‐end grocery stores). Following a 2‐week period of flyer distribution, data collection occurred over a 1‐year period from November 2022 until November 2023. Halfway through the data collection period, a second wave of flyer distribution was performed to ensure that community members had longitudinal survey access.

To be included in the study, participants must have responded that their sex assigned at birth was female. Additionally, participants must have been age 18 or older at the time of the survey to participate. A statement was provided in the beginning of the survey to confirm informed consent.

### 2.2 Statistical Analysis

When identifying respondent compliance, national guidelines were used. The ACOG advises female individuals to have a clinical breast examination every 3 years between the ages of 25 and 39 and within the last year when aged 40 and older [6]. ACOG guidelines for screening mammography were also used, indicating a need for biannual screening mammography for all female individuals between the ages of 40 and 75 [6].

Descriptive statistical analysis was performed on all data addressing participant age, household income, education level, ethnicity, language, screening compliance, and perceptions. Odds ratios were performed to identify disparities in screening mammography and clinical breast examinations between demographics. Additionally, respondent perceptions of mammography and clinical breast examination were analyzed and compared via descriptive statistics assessing percentage of respondents selecting each sentiment regarding the screenings. This was performed on the following software: IBM SPSS, Python, and Pandas library.

## 3 Results

### 3.1 Screening Mammography

A total of 28 individuals completed the mammography portion of the survey and were eligible for inclusion in the study (Table 1). Twenty‐two respondents (78.57%) completed the survey in English, whereas six (21.43%) completed the survey in Spanish. The mean age of respondents was 47.68 years (standard deviation [SD] = 8.17), and mean household income was 43,660.71 United States Dollars (USD) (SD = 50,829.97). Most respondents identified high school as their highest level of education (*n* = 12; 43%), and the majority reported their ethnicity as Hispanic or Latino (*n* = 16; 57%).

**TABLE 1:** Demographic distribution of survey respondents for screening mammography.

Age	40–44 years old	45–54 years old	55–65 years old	Over 65 years old				
Number of individuals	18	2	8	0				
Household income	$10,000 or less	$11,000–$40,000	$41,000–$50,000	$51,000–$70,000	$71,000–$90,000	$91,000–$110,000	$110,000–$199,000	$200,000 or more
Number of individuals	8	11	2	2	1	1	2	1
Highest level of education	Did not complete elementary school	High school	Some university/college	Graduated university	Masters degree	Doctoral degree		
Number of individuals	2	12	7	6	0	1		
Ethnicity	Black or African American	Hispanic or Latino	Native American or Alaskan	White or Caucasian	“Other”			
Number of individuals	4	16	0	7	1			

A total of 24% (*n* = 6) had never had a mammogram, 12.5% (*n* = 3) had a mammogram in the past but were overdue at the time of survey completion, and 57.1% (*n* = 16) were up to date on screening mammography.

Individuals of Hispanic or Latino race were at 2.72 times greater odds of never having had a mammogram (95% confidence interval [CI] [0.26, 29.07]) and at 2.57 times greater odds of being overdue for screening mammography (95% CI [0.21, 31.71]) in comparison to those with White or Caucasian race. Respondents with an income of 40,000 USD or lower were 3.08 times more likely to have never had a mammogram (95% CI [0.30, 31.33]) and 1.55 times more likely to be overdue for screening mammography (95% CI [0.17, 20.86]) in comparison to those with an income level of greater than 40,000 USD. Those with a highest level of education being high school or lower were 6.7 times more likely to have never had a mammogram (95% CI [0.66, 67.47]) and 4.4 times more likely to be overdue for screening mammography (95% CI [0.32, 60.62]).

Barriers to screening mammography were reported as cost (48%, *n* = 12), lack of knowledge (20%, *n* = 5), lack of time (12%, *n* = 3), transportation (8%, *n* = 2), language barriers (4%, *n* = 1), and “other” (25%, *n* = 6). For those who selected other, the following responses were manually input: none (*n* = 3), no health insurance (*n* = 2), did not specify (*n* = 1).

### 3.2 Clinical Breast Examination

A total of 59 individuals aged 21 and older completed the survey and were included in the clinical breast examination portion of the study, as demonstrated in Table 2. A majority (*n* = 52; 88.13%) of respondents completed the survey in English, whereas seven (11.87%) completed the survey in Spanish. The mean age of respondents was 38.38 years (SD = 10.50) and mean household income was 44,974.57 USD (SD = 49,180.39). Most respondents reported high school as their highest level of education (*n* = 19; 32%) and Hispanic or Latino as their race (*n* = 31; 53%).

**TABLE 2:** Demographic distribution of survey respondents for clinical breast examination.

Age	Less than 21 years old	21–39 years old	40–44 years old	45–54 years old	55–65 years old	Over 65 years old		
Number of individuals	0	31	18	2	8	0		
Household income	$10,000 or less	$11,000–$40,000	$41,000–$50,000	$51,000–$70,000	$71,000–$90,000	$91,000–$110,000	$110,000–$199,000	$200,000 or more
Number of individuals	15	22	4	8	3	2	2	3
Highest level of education	Did not complete elementary school	High school	Some university/college	Graduated university	Masters degree	Doctoral degree		
Number of individuals	2	19	16	15	5	2		
Ethnicity	Black or African American	Hispanic or Latino	Native American or Alaskan	White or Caucasian	“Other”			
Number of individuals	10	31	1	13	4			

A total of 22% (*n* = 13) had never had a clinical breast examination, 27.1% (*n* = 16) had a clinical breast examination performed previously but were overdue at the time of survey completion, and 50.8% (*n* = 30) were up to date on their clinical breast examination.

Respondents reporting Hispanic or Latino race were 5.71 times (95% CI [0.65, 50.28]) more likely to have never had a breast examination and 7.7 times (95% CI [0.80, 74.05]) more likely to be behind on breast examination than individuals of Caucasian race. Respondents with an income of 40,000 USD or lower were 2.35 times more likely to have never had a clinical breast examination (95% CI [0.57, 9.68]) and 3.06 times more likely to be overdue (95% CI [0.54, 17.23]). Respondents who reported their highest level of education being high school or lower were at 1.17 times greater odds (95% CI [0.33, 4.18]) of never having had a clinical breast examination and 4.10 times greater odds (95% CI [0.86, 19.61]) of being overdue for clinical breast examination.

The self‐reported barriers to clinical breast examinations were cost (*n* = 25; 42.4%), lack of knowledge (*n* = 19; 32.2%), lack of time (*n* = 16; 27.1%), transportation (*n* = 6; 10.2%), language barrier (*n* = 2; 3.4%), and an unspecified “other” (*n* = 11; 18.6%).

### 3.3 Perceptions

Most (*n* = 37; 67.3%) respondents reported a strong interest in preventative women's health services, 30.9% (*n* = 17) had moderate interest, and 1.8% (*n* = 1) had little interest. No participants reported having no interest.

A third (*n* = 18; 32.7%) of respondents endorsed seeing a healthcare provider less than once a year, whereas 30.9% (*n* = 17) of participants saw a healthcare provider once per year, 20.0% (*n* = 11) two to three times per year, and 16.4% (*n* = 9) reported seeing a healthcare provider four or more times per year. Half (*n* = 28; 50.9%) of respondents reported that they would like to see a healthcare provider for women's health services, 47.3% (*n* = 26) would like to see a healthcare provider for both women's health services and for other health conditions, and 1.8% (*n* = 1) would like to see a healthcare provider solely for conditions not relating to women's health.

**FIGURE 1:**
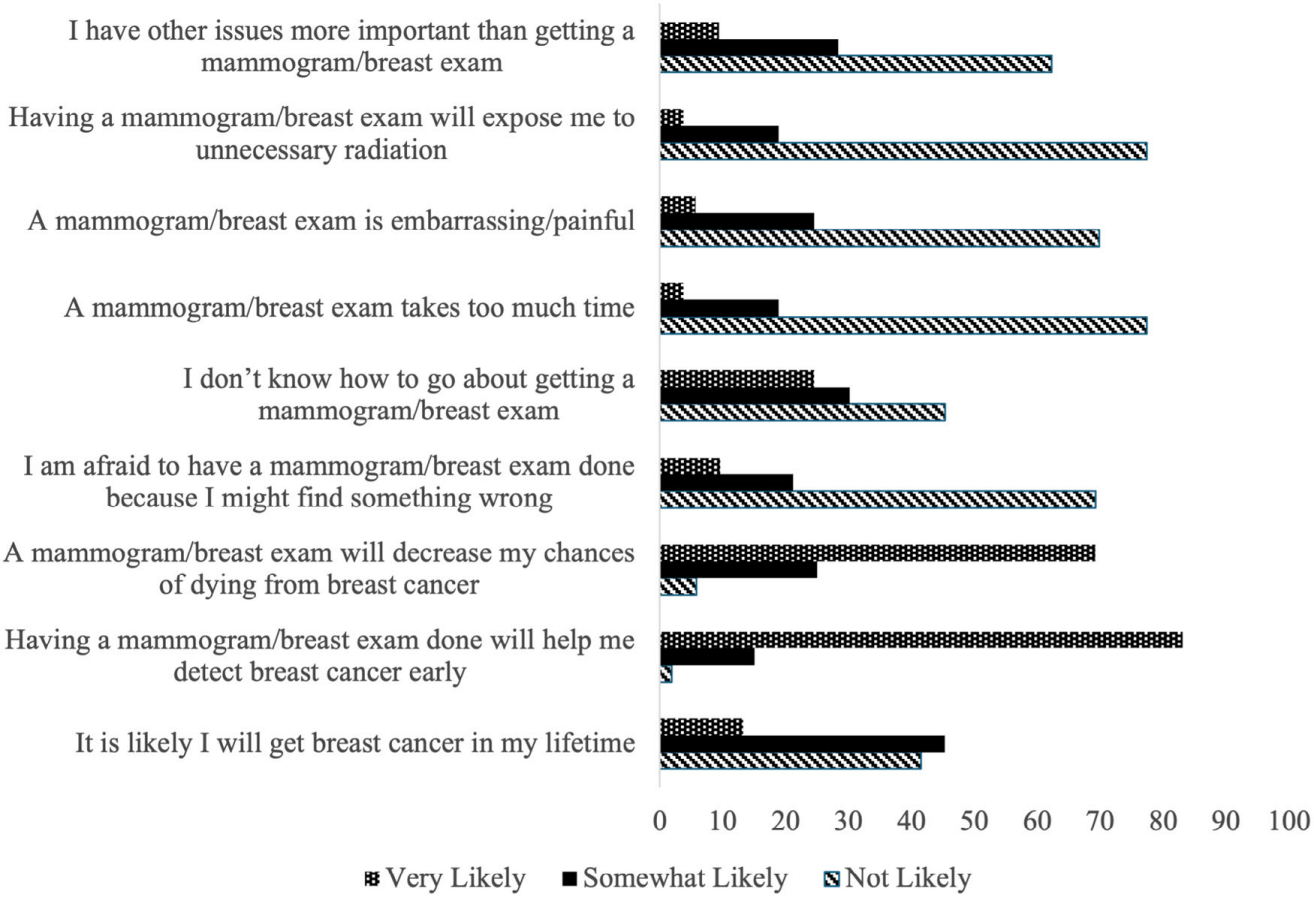
Percentage of respondents who endorsed their perceptions on various sentiments relating to breast cancer.

Over half (*n* = 31; 56.4%) of respondents selected that they believe it is very likely or somewhat likely that they will develop breast cancer in their lifetime (Table 3). Additionally, 72.7% (*n* = 40) believed that screening mammography and clinical breast exams are very likely to help detect breast cancer early, and 69.1% (*n* = 38) believe that these tests are very likely to decrease chances of dying from breast cancer. A total of 20 respondents (37%) admitted that they are afraid or somewhat afraid of mammography and clinical breast examination because they might reveal a medical issue. A total of 14 respondents (25.5%) stated that they do not know how to go about scheduling a mammogram/clinical breast examination, whereas 7.3% (*n* = 4) believed that these screenings take too much time, and 29.1% (*n* = 16) believed that they are embarrassing and painful or somewhat embarrassing and painful. Nearly a third (*n* = 16; 29.1%) of respondents believe that it is very likely or somewhat likely that screening mammography exposes patients to unnecessary radiation, whereas 34.5% (*n* = 19) report that they have other issues more important than screening mammography or clinical breast examination. These findings are visualized in Figure 1.

**TABLE 3:** Percentage of responses for breast cancer screening sentiments.

	Not likely	Somewhat likely	Very likely
It is likely I will get breast cancer in my lifetime	41.51	45.28	13.21
Having a mammogram/breast exam done will help me detect breast cancer early	1.89	15.09	83.02
A mammogram/breast exam will decrease my chances of dying from breast cancer	5.77	25	69.23
I am afraid to have a mammogram/breast exam done because I might find something wrong	69.23	21.15	9.62
I don't know how to go about getting a mammogram/breast exam	45.28	30.19	24.53
A mammogram/breast exam takes too much time	77.36	18.87	3.77
A mammogram/breast exam is embarrassing/painful	69.81	24.53	5.66
Having a mammogram/breast exam will expose me to unnecessary radiation	77.36	18.87	3.77
I have other issues more important than getting a mammogram/breast exam	62.26	28.3	9.43

## 4 Conclusion

This study not only yields critical local insights but also offers a lens through which to examine broader national trends and disparities. Comparing our findings with existing national data allows us to assess how North Texas fares relative to other regions and provides valuable context for understanding the implications of screening underutilization. Examining the repercussions of inadequate screening uptake unveils sobering statistics regarding breast cancer outcomes.

Previously conducted national studies consistently demonstrate that delayed diagnosis due to underutilization of preventive screenings significantly diminishes treatment efficacy and survival rates. In the United States in 2015, breast cancer was the leading cause of death among Hispanic women [12]. This population is nationally recognized for receiving guideline‐adherent screenings at lower rates than non‐Hispanic White women [12]. In the current study conducted over 2022 to 2023 in Fort Worth, Texas, it was also revealed that Hispanic or Latino women had 2.72 times greater odds of never having had a mammogram and 2.57 times greater odds to be overdue for screening mammography in comparison to those of White or Caucasian race. This stark reality underscores the urgency of addressing barriers to screening access and adherence, particularly among vulnerable populations. Fort Worth's demographic composition, closely mirroring national averages in terms of racial and financial distribution, renders our findings highly generalizable to the broader US population. This demographic alignment enhances the relevance and applicability of our study's insights in informing nationwide strategies aimed at improving healthcare access and mitigating disparities.

A unique aspect of this study lies in the inclusion of data on participant perceptions and sentiments, shedding light on the intricate interplay between attitudes, beliefs, and screening behaviors. Our results revealed that although a majority of participants understood the ability for breast cancer screenings to detect cancer early and reduce morality, 37% were afraid of their ability to reveal that something is wrong. Additionally, 25.5% reported that they did not know how to schedule a mammogram or clinical breast examination. Addressing these perceptual barriers necessitates innovative approaches grounded in evidence‐based practices. Strategies such as culturally sensitive education campaigns, community outreach initiatives, Non‐English Language Preference (NELP) education initiatives for clinicians and advocacy staff, as well as overall communication enhancements, are potential options. Many of these avenues have shown promise in mitigating fears, dispelling myths, and fostering positive attitudes toward preventive screenings. Regarding cultural competency to improve patient perceptions, the embedding of Community Health Workers (CHW), or frontline public healthcare workers, has been shown to lower healthcare costs through direct communication and effective education of their communities [13].

This study contributes to a comprehensive understanding of women's preventive healthcare utilization by elucidating local disparities, contextualizing findings within national trends, and unveiling the role of perceptions in shaping screening behaviors. By leveraging these insights, policymakers and healthcare stakeholders can utilize these findings to develop targeted interventions aimed at reducing barriers, enhancing screening uptake, and ultimately improving breast cancer outcomes for all women in the nation.

This study is the first to combine patient compliance, barriers, and subjective perceptions regarding breast cancer screenings. Strengths of this study include the year‐long data collection period as well as the focus on low‐income areas to best identify barriers for historically underserved populations. Availability of the survey and flyers in both English and Spanish encouraged Spanish‐speaking respondents to be included in the study. Additionally, Fort Worth and the greater Fort Worth area are demographically distributed similarly to the United States. A limitation to the present study is a relatively small sample size, which reduced power and weakened CIs on statistical analysis. Additionally, respondents were required to have a phone and internet access given the use of flyers with a QR code for survey distribution. Similar studies including greater populations and multiple cities may allow for stronger results and suggestions for intervention.

Overall, Hispanic, lower income, and less educated individuals are more likely to have inadequate breast cancer screening. This is due to several barriers including cost, lack of knowledge, and lack of time. These discrepancies are present despite a majority of respondents expressing interest in obtaining women's healthcare, believing that they are effective in detecting breast cancer and decrease risk of dying from breast cancer. We suggest that the previously mentioned demographics be targeted in campaigns to improve access and education surrounding breast cancer screenings. This study would benefit from further data collection and expansion to multiple cities in the United States.

## Author Contributions

**Sofia Eva Olsson:** conceptualization, data curation, investigation, methodology, project administration, resources, writing of original draft, review and editing. **Sameep Shah:** data curation, formal analysis, methodology, review and editing. **Erin Haase:** investigation, writing of original draft, review and editing. **Emma Butler:** investigation, writing of original draft, review and editing. **Isabella Amado:** investigation, review and editing. **Kelly Pagidas:** conceptualization, supervision, methodology, review and editing.

## Ethics Statement

This study was submitted to and approved by the Texas Christian University Institutional Review Board with the identification number 2022‐149.

## Consent

Participants gave informed consent for participation in the study.

## Conflicts of Interest

The authors declare no conflicts of interest.

## Data Availability

The data that support the findings of this study are available from the corresponding author upon reasonable request.
